# Analysis of energy storage materials for developments in solar cookers

**DOI:** 10.12688/f1000research.126864.1

**Published:** 2022-11-11

**Authors:** Rahul Khatri, Rahul Goyal, Ravi Kumar Sharma

**Affiliations:** 1 Mechanical Engineering Department, Manipal University Jaipur, Jaipur, Rajasthan, 303007, India

**Keywords:** Solar Energy, Solar Cooker, Energy Storage, Sensible Heat, Latent Heat, Storage Capacity, Thermal Performance and Phase Change Materials

## Abstract

Solar energy is accessible freely and can be utilized for many household and industrial applications. The consumption of solar energy for cooking applications has found significant success. Various innovations have been employed in facilitating cooking during off-sunshine hours. Thermal energy storage helps in overcoming the fluctuations in the supply of energy required for cooking during different time periods of the day. This study focuses on the different types of thermal energy storage mediums that are currently utilized in solar cooking. Primarily, oils and pebbles are most commonly used as sensible heat storage (SHS) while organic phase change materials (PCMs) are used as latent heat thermal energy storage materials (LHTES).

The properties and performances of various SHS and latent heat storage (LHS) mediums have been compared for their suitable utilization. SHS materials are cost-effective but have lower thermal gradient compared to LHTES materials. The energy storage capability of LHTES is high while degradation with the increasing number of charging and discharging cycles is also considerable. The melting point should be close to the utilization temperature for being used as LHTES as thermal diffusivity of the materials greatly influences the performance of solar cookers. The cooking time is lower for solar cooking systems equipped with energy storage compared to non-equipped cooking systems. It is recognized that the use of energy storage has been proved as a huge advantage to solar cooking systems, however, the design, and heat transfer characteristics of the cooking vessel along with the storage material type and volume must be optimized in order to make this technology more influential.

## Introduction

The future belongs to renewable energy, as the demand and supply gap of fossil fuel is increasing day by day. Solar energy seems to have the potential to cater to the energy needs of the world as they have grown strongly over the last few decades in terms of energy collected. Solar thermal energy systems have higher efficiencies, but they lack in the aspect of energy backup. Many applications of these systems require a continuous supply of energy, but the unavailability of energy during off-sunshine hours limits their use. Energy storage systems bridge this gap and maintain the required energy supply, which increases the performance and reliability of these systems.

Solar energy is one of the simplest green energy options to meet the cooking energy needs. It not only reduces health hazards caused by the use of traditional fuels but it is also eco-friendly. Two types of commonly used direct solar cookers are box type and concentrating type. These may have reflectors for enhancing optical performance or can work without reflectors. In indirect-type solar cooking arrangements, the system may be integrated with flat plate collector (FPC), evacuated tube collector (ETC) or with a concentrating collector. ^1^

The social importance of solar cookers was explored by Escobar. ^2^ The unavailability of energy during off-sunshine hours is its major drawback. Solar cookers with thermal energy storage (TES) are a good alternative to the traditional cooking methods and also reduce CO _2_ emissions in a significant manner. ^3^

Solar energy systems will be highly productive and reliable with the integration of energy storage mediums. ^4^^–^^6^ Use of TES enhances the performance with low running and operating expenses. This also permits the use of solar cooking systems during off-sunshine hours.

Use of energy storage in solar energy systems improves their efficiency and reliability. For high temperature applications, phase change materials (PCM) are most useful, however, their economic feasibility is still unattended. As suggested by Gautama *et al*., ^7^ sensible heat storage (SHS) combined with PCM can improve the performance of energy storage systems.

Utilization of water and dodecanoic acid as energy storage in solar collector was carried out by Joseph *et al*. ^8^ Water has high charging and discharging rates but low storage capacity while LHES was reported to be potentially better for reducing energy storage volumes.

The properties of a good TES medium are, melting point of PCM should be same as the working temperature, high energy storage density, high latent heat, and high heat capacity. These are required to have high thermal conductivity for faster heat transfer rates. ^9^ High mass to volume ratio and high specific heat are two common desirable properties of energy storage mediums while working with solar energy systems.

## Methods

This paper categorizes various research works carried out on solar cooking with thermal energy storage mediums. Classification of thermal energy storage medium along with a discussion on sensible heat storage mediums and latent heat storage mediums is presented. The advantages of SHS and latent heat storage (LHS) mediums for suitability in solar cooking applications are also reviewed. Various thermo-physical properties of these materials are also discussed to analyze their use as per the end-user needs.

### Classification of thermal energy storage materials

**Figure 1.**
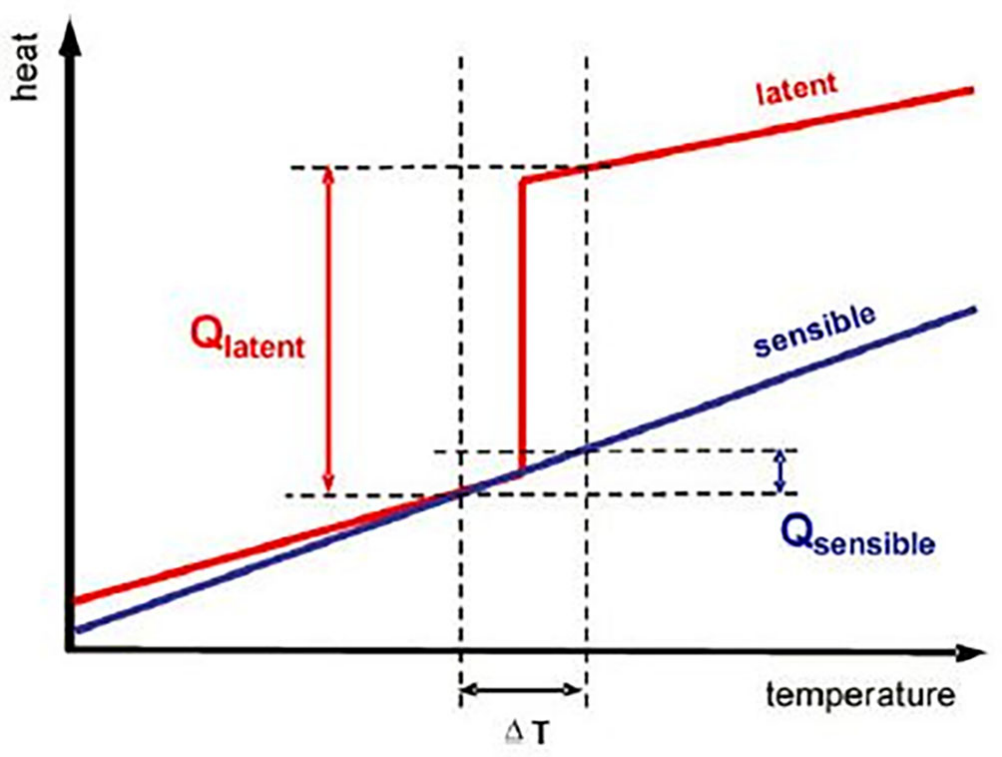
Comparison between latent heat storage (LHS) and sensible heat storage (SHS). ^10^

**Figure 2.**
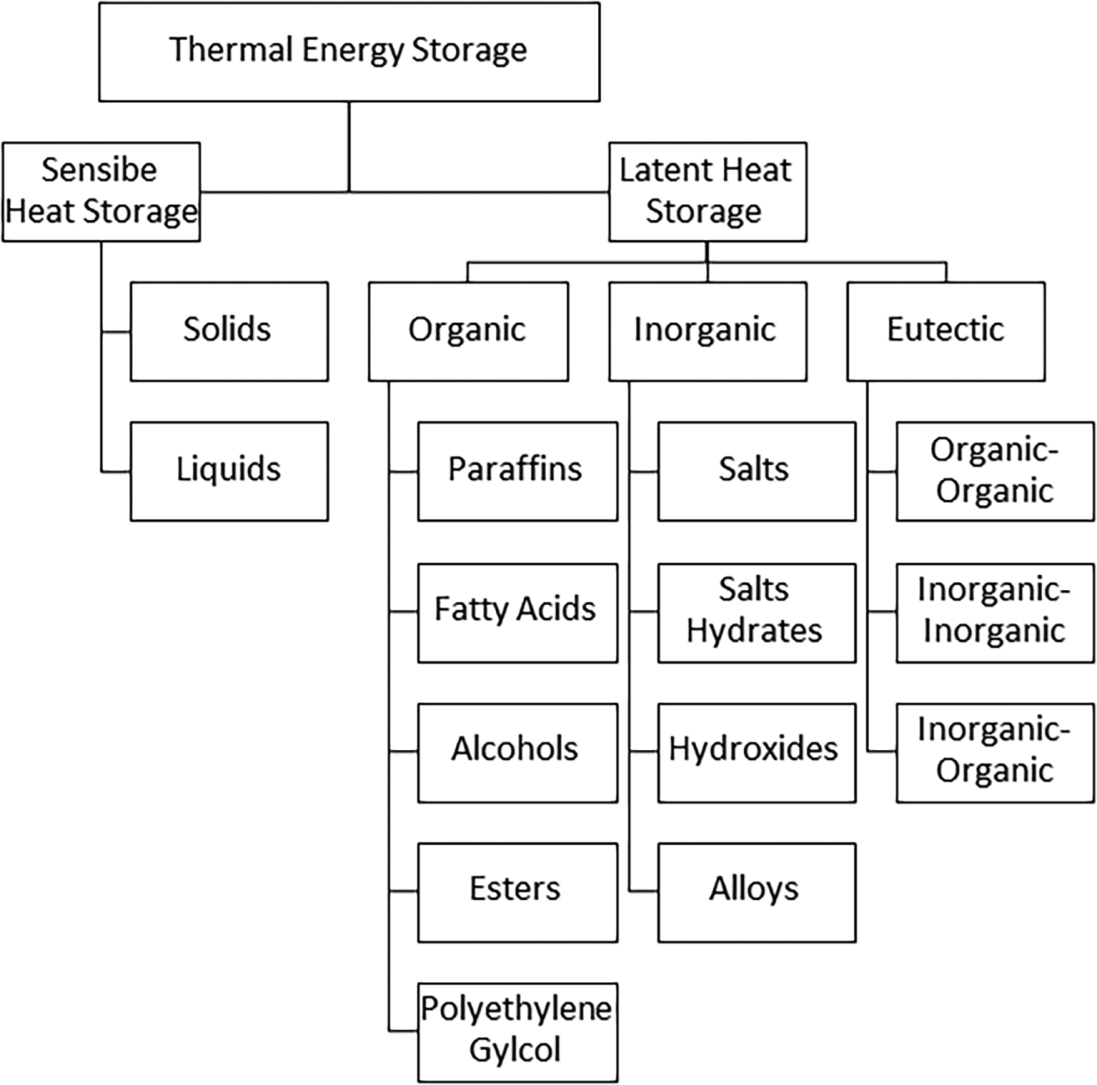
Classification of thermal energy storage (TES) mediums.

The storage of energy during the daytime for supply during off-sunshine hours is possible if it is stored in one or other form. TES can be classified under SHS and LHS mediums. SHS mediums do not have a change in their physical state while LHS mediums absorb energy, converting their physical state from solid to liquid, and from liquid to vapor. The heat and temperature variations in LHS and SHS have been presented in Figure 1. The detailed classification of energy storage mediums is presented in Figure 2.

The heat storage capacity of latent heat thermal energy storage (LHTES) mediums is high as compared to SHTES medium. As the latent heat of fusion (Q _latent_) does not have any temperature change, the charging and discharging include both temperature change and phase change in LHTES.

### Energy storage mediums in solar cooking

***SHS mediums***

SHS mediums store energy in the form of heat creating temperature difference. Materials having a high specific heat are more useful as SHS as they can store higher energy for a unit change of temperature difference. They are relatively cheaper and easily available as compared to other materials, which increase their usefulness.

SHS mediums can be in solid or liquid form. In liquid, water is used in many applications as it is cheaply available and has high specific heat. For high temperature applications, various SHS liquid mediums like oils, liquid metals, or salts can be used. In a few articles, ^11^^,^^12^ working of SHS mediums to up to 180°C has been reported, but the increase in the total weight of the system is a concern with these mediums. Economic analysis for different solar concentrating devices used in cooking applications has been carried out by Widjaja *et al*., ^12^ with SHS medium, the payback period for solar cooking system was half of the payback period without SHS.

Saxena *et al*. ^13^ experimentally investigated the use of grainy carbon powder as an SHS medium in box-type solar cookers (BTSC); the best output was reported with a composite prepared with LHS i.e. paraffin wax. The use of palm oil as an energy storage medium was studied ^14^ and it was reported that the heating efficiency was doubled with the use of energy storage mediums. A low cost SHS medium was prepared with sand and carbon for testing with solar cooker. ^15^ The cooker was found viable for off-sunshine hours cooking with an efficiency of around 37%.

**Figure 3.**
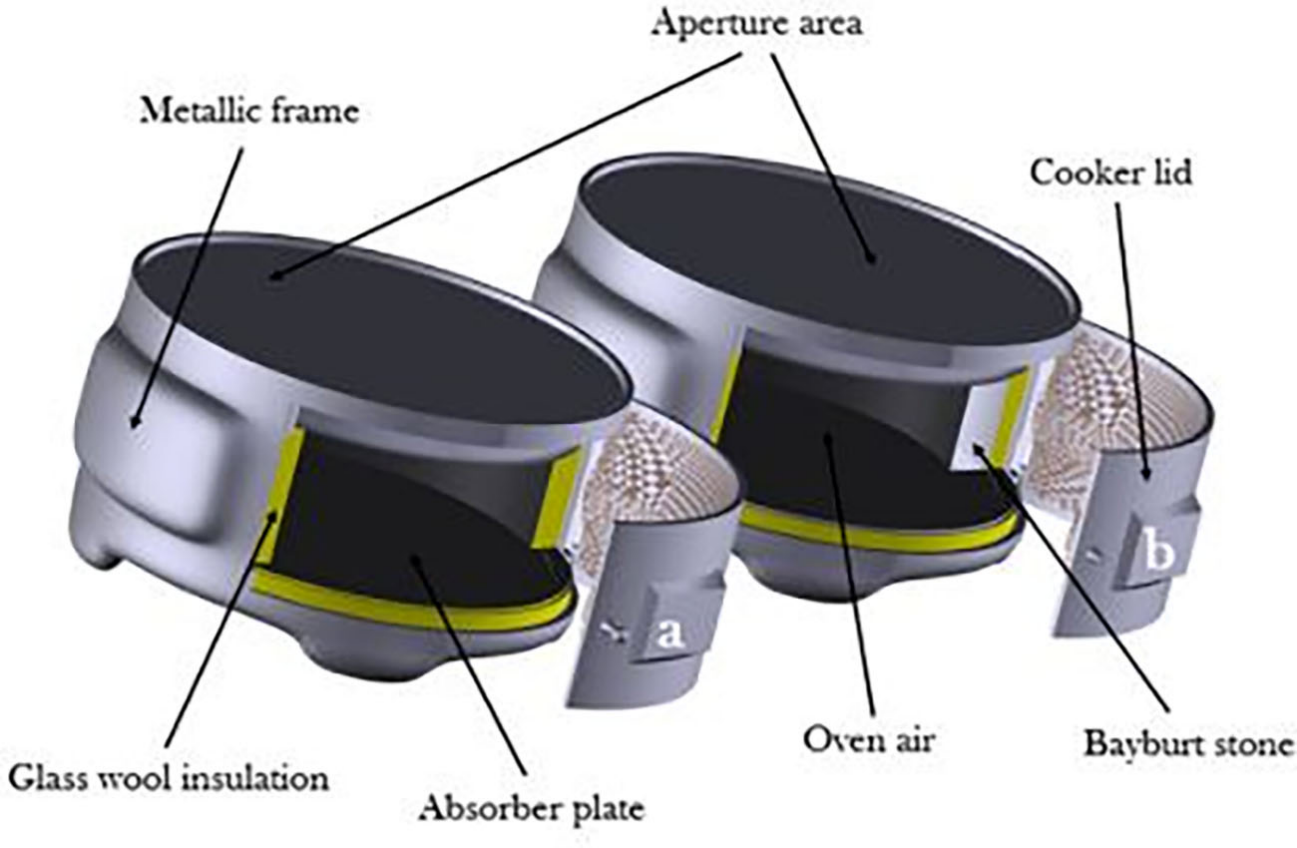
Schematic of conventional and sensible heat storage (SHS) integrated solar cookers. ^16^

Use of bayburt stone as an SHS medium in BTSC as shown in Figure 3 resulted in efficient cooking during off-sunshine hours. The efficiency improvement of about 40% was achieved with this SHS medium. ^16^ The temperature in solar cooker with Lapland granite rocks used as SHS during experimentation was maintained above 40°C for almost 225 minutes, while without SHS material, it was above 40°C for only 45 minutes. The author ^17^ concluded that, when using TES, the cooking capability of the solar cookers can be extended for a longer duration.

An oil-pebble bed-based energy storage medium for solar cookers was studied by Mawire *et al*. ^18^ Methods of charging and discharging the energy storage medium also play an important role in its performance. Engine oil as an energy storage medium with BTSC systems was studied by Nahar. ^19^ A temperature difference of 23°C was achieved with energy storage compared to a system without energy storage. Food was cooked perfectly during off-sunshine hours where it was not cooked without energy storage medium. The various materials used as SHS medium in solar cooking system along with their thermal and physical properties are presented in Table 1.

**Table 1.** Sensible heat storage (SHS) mediums for solar cooking systems.

S.No.	Cooker type	Material	Density kg/m ^3^	Sp. heat (kJ/kg-K)	Melting point °C	Thermal conductivity kJ/kg-K	Thermal diffusivity m ^2^/s/10 ^6^	Ref.
1	Box	Grainy carbon powder (GCP)	460	0.93		0.11	10.2	^13^
2	Box	Vegetable oil (Palm oil)	915	1.7	24			^14^
3	Box	Sand	1450	0.80		0.26	0.35	^15^
		Granular carbon	460	0.93		0.11	1.02	
4	Box	Bayburt Stone	2370	0.7144		0.59		^16^
5	Box	Lapland granite rocks	2092	0.543				^17^
6	PDC	Pebbles	2801	0.745		1.8		^18^
7	Box	Engine oil	850	1.76		0.149		^19^

***LHS mediums***

LHS mediums generally have a phase change during energy interactions. During energy exchange, i.e. charging and discharging, the energy can be absorbed in raising the temperature as well as changing the physical state. They are also referred to as PCM. It is desirable to use LHS mediums where uniform temperature needs to be maintained for a longer duration, however, PCM increases the initial cost of the system. ^20^ Chemically, the PCMs must be stable, compatible with materials, should be non-toxic and non-flammable. ^21^

Organic PCMs are the most commonly used energy storage medium in solar cooking. Nitrate salts are mostly used in high temperature applications, while for low and moderate temperatures, organic materials are used. The systems with working temperatures lower than 120°C do not effectively utilize the latent heat storage mediums. ^22^ The various materials used as LHS medium in solar cooking system along with their thermo-physical properties are presented in Table 2.

**Table 2.** Latent heat storage (LHS) mediums for solar cooking systems.

S.No.	Cooker type	Material	Density (kg/m ^3^)	Sp. heat (kJ/kg-K)	Storage capacity (kJ/kg)	Melting point (°C)	Latent heat (kJ/kg)	Thermal conductivity (W/m-K)	Ref.
1	Box	Paraffin wax	800	2	250	41-44		0.2	^13^
2	Parabolic dish solar collector (PDSC)	Paraffin	852			80	220		^26^
3	Box	Erythritol	1480-S 1300-L	1.383-S 2.76-L		117.7	339.8		^24^
4	Box	Benzoic acid	1270	1.2		121.7	142.8		^14^
		Stearic acid	847	1.8		55.1	160		
5	PDSC	Magnesium chloride hexahydrate	1560	1.72-S 2.82-L		118	167	0.57	^33^
6	PDSC	Solar Salt *	1800			210-220	108.67	0.8	^27^
7	Box	Acetanilide		2		118.9	222		^25^
8	Box	Solar salt mixture **		1.441		145.14	101.5		^31^
9		Magnesium nitrate hexahydrate #				89	134		^34^
10		Galactitol				187-191			^28^
11	Parabolic solar collector (PSC)	Stearic acid		1.590		55-70	155		^29^
12	PSC	Erythritol	1480-S 1300-L	1.38-S 2.76-L		118	340	0.733-S 0.326-L	^30^
		Paraffin	880-S 770-L	1.8-S 2.4-L		100	140	0.21-S 0.2-L	
13	Box	Paraffin wax					210		^2^
14	Box	Commercial grade acetamide	1159-S 998-L	1.94		82	263		^35^

* Mixture of sodium nitrate and potassium nitrate.

** Mixture of NaNO _2_, NaNO _3_, and KNO _3_.

^#^ (Mg (NO _3_) _2_ 6H _2_O).

*Use of organic PCM*

Organic PCMs are a group of paraffins and non-paraffin materials. Fatty acids, alcohols, glycols and esters are classified under non-paraffin phase change materials. They have low thermal conductivity in solid phase and are relatively cheap. Paraffin wax is the most commonly used organic PCM as it has comparable thermal properties, i.e., high density and high latent heat.

The cooking efficiency up to 54% can be achieved and cooking time reduced with the use of paraffin as an energy storage medium in BTSC. It was concluded by Saxena *et al*. ^13^ that the composite of SHS and LHS performed better than the SHS and LHS individually. The cooking time was reduced by one hour when paraffin wax used as an energy storage medium. ^23^ Comparative studies between solar cooker with and without energy storage was reported by Reddy *et al*. ^2^ Paraffin PCM was used and a temperature difference of 17 °C was maintained during evening time in the cooker with TES compared to the cooker without TES.

Use of erythritol as an energy storage medium was investigated ^24^ using a box-type solar cooker and it was reported that both the charging and discharging time was increased using PCM. Thermal stability of the system was better with PCM during off-sunshine hours. Use of acetanilide as PCM with box-type solar cooking system was investigated by Buddhi *et al*., ^25^ showing high temperature for well-cooked food. Use of benzoic acid and stearic acid as LHS medium was studied by Adetifa *et al*. ^14^ and it was concluded that the high temperature of water was realized with energy storage, i.e., 80°C, and 50°C was maintained without solar radiations.

Solar cooker with PDC was studied by Senthil ^26^ using paraffin wax as an energy storage medium. Heating time for water was reduced to 90 min compared to 120 min without PCM. Improved productivity and thermal performance of the cooker were observed with PCM. Solar cooking using solar salt as PCM for high temperature application was carried out by Bhave *et al*. ^27^ An indoor experiment was carried out and 170–180°C was easily achieved with this system. The device was found convenient for cooking inside the kitchen and with minor geometrical modifications it can serve different end user needs.

Galactitol as PCM was utilized for medium temperature solar cooking applications by John *et al.* ^28^ The lifespan of Galactitol as PCM was reported to be less than 100 days if utilized every day for cooking application. Stability at high temperatures of 150°C was reported, but due to the short life span, the author concluded that it is unstable for energy storage applications.

**Figure 4.**
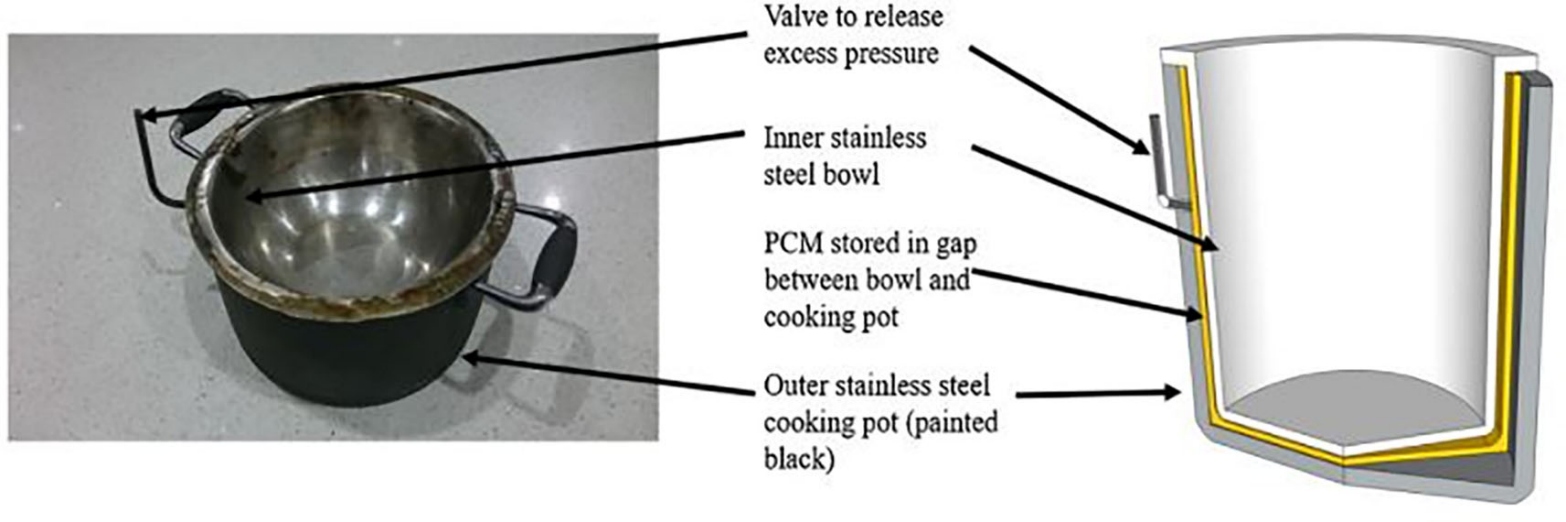
Phase change material (PCM) integrated cooking pot. ^29^

Stearic acid as a PCM using a collapsible parabolic solar cooker was studied by Keith *et al*. ^29^ PCM integrated cooking pot is shown in Figure 4 which was used to cook food and subsequently kept it warm for longer duration. The payback period of the developed solar cooking system was found to be less than 53 weeks.

Paraffin and erythritol were used as energy storage mediums in a parabolic concentrating- type solar cooking system. Lecuona *et al*. ^30^ concluded that the use of PCM and insulating the utensil after energy storage allows cooking of dinner and next day breakfast. The study also concluded that higher latent heat and conductivity of erythritol are advantageous for faster indoor cooking.

*Use of inorganic PCM*

Inorganic PCMs are salts, salt hydrates and metal alloys. Salts and salt hydrates have the highest latent heat to volume ratio, thermal conductivity and smooth phase transition. Drawbacks of inorganic salts as reported by Khan *et al*. ^20^ are change of volume, low thermal conductivity and high cost.

Solar salt was used as an energy storage medium with a box-type solar cooker by Coccia *et al*. ^31^ It was concluded that thermal stability was improved and heat retention was increased 1.86 times than that without a storage medium. Mussard *et al*. ^32^ concluded that the use of inorganic PCM like nitrate salt has potential of replacing traditional cooking methods. These systems are slower for traditional cooking but the quality of heat transfer is better in these systems.

**Figure 5.**
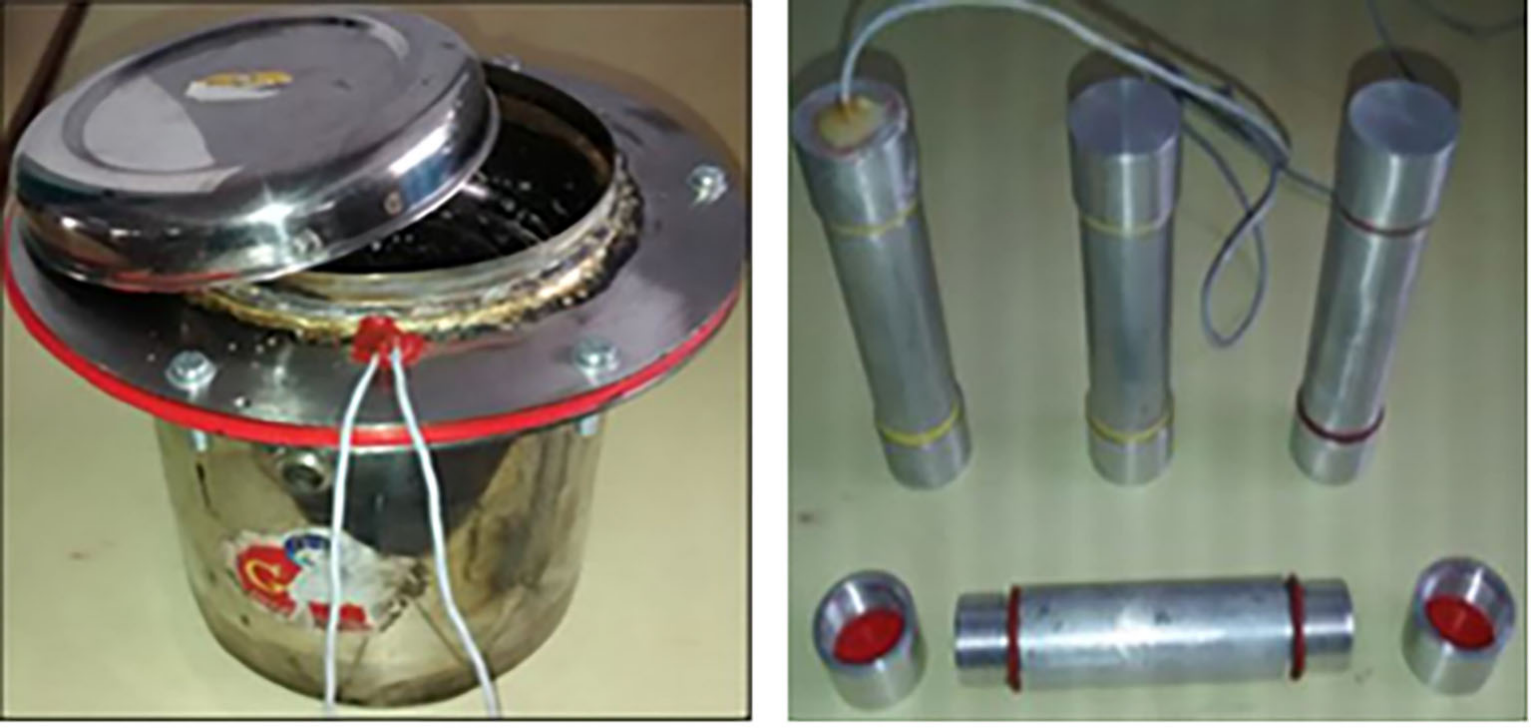
Storage cooker and aluminium tubes. ^33^

Magnesium chloride hexahydrate was used as PCM with parabolic dish solar collector (PDSC) by Bhave *et al*. ^33^ The cooker with PCM tubes is presented in Figure 5. The PCM was heated upto 130°C i.e., 12°C above the melting point of the PCM. 32.66% utilization was achieved by the developed system.

Magnesium nitrate hexahydrate was used as PCM for indirect solar cooking unit by *Hussein et al.* ^34^ The developed cooker was found suitable for heating or keeping the food hot during off-sunshine hours till the next morning. Box-type solar cooker incorporating acetamide as PCM was experimentally compared with solar cooker without PCM by Sharma *et al*. ^35^ The results concluded that cooking during off-sunshine hour is possible with cooker incorporated with PCM. Due to heavy weight, metallic PCMs are rarely considered as an energy storage medium in solar cooking.

***Heat transfer fluids (HTFs)***

Solar energy systems while having energy concentration can store thermal energy in direct or indirect cooking arrangements. HTFs are used to transfer the heat from the source to the indirect cooking system. The heat transfer fluid, while having a major role in transferring the heat, can also behave as an energy storage medium. Few articles suggested that the storage medium can also be used as an HTF. ^36^ The high specific heat and thermal conductivity appraise it to behave as energy storage and transfer medium. Low viscosity for minimum pumping power is desirable for heat transfer fluids. Table 3 lists HTFs used in solar cooking and their properties.

**Table 3.** Heat transfer fluids (HTFs) for solar cooking systems.

S.No.	Cooker type	Material	Density (kg/m ^3^)	Sp. heat (kJ/kg-K)	Flash point °C	Thermal conductivity kJ/kg-K	Ref.
1	PDSC	Therminol (Taurus therm 500)	840	3.22	220	0.11	^27^
2	PDC	Oil	869	1.89	225	0.143	^18^
3	PDC	Therminol-55 (Synthetic Oil)					^37^
4	PTC	Therminol-55	875		193	0.1276	^39^

Use of therminol-55 as an HTF for energy modelling of cooking systems was carried out by Bade *et al*. ^37^ and proposed this for water scarce regions. It also enhances the heat transfer capability and has a very minimal payback period. Therminol-55 (synthetic oil) was used as heat transfer fluid by Singh ^38^ for realizing indoor cooking using solar energy. Vegetable oil was used as HTF in solar cooking system for big families and positive results have been obtained by Schwarzer *et al*. ^11^

## Discussion and conclusions

Solar cooking can be a potential replacement for traditional cooking systems. This will lead to environmental, economic and health benefits to the end user. Use of solar cookers in conjunction with energy storage mediums to enhance their performance is analyzed for its use during off-sunshine hours. The role of energy storage materials, their importance and advantages in solar cooking application are discussed in detail.

Solar cooking systems using both SHS and LHS have been reviewed and discussed. Use of SHS found limited applications, however, optimized used of these mediums resulted in better thermal output. Pebbles are most commonly used SHS as they have comparable thermal properties, are readily available and are available free of cost.

Most of the studies reviewed used LHTES mediums with solar cooking. The results suggested promising improvement in the performance of solar cooking especially during off-sunshine hours. With moderate melting point and high latent heat of fusion, paraffin wax and erythritol are the most commonly used latent heat storage mediums.

Heat transfer fluids are generally used to integrate the direct and indirect cookers. The feasibility of indoor cooking has been found in a few studies where HTF is highly recommended. Therminol-55 is the most commonly used HTF.

The following conclusions can be drawn from the present study:

• Box-type and parabolic dish-type solar cooker are commonly used solar cooking devices.
• The use of energy storage mediums shows remarkable progress in the thermal output of solar cooking systems.
• Working of solar cooker during evening and till next morning has been found feasible with the use of energy storage.
• Thermal performance improvement with PCM is high whereas economically SHS was found more viable.
• Use of inorganic PCMs i.e. salts and salt hydrates are associated with drawbacks like corrosion, poor heat transfers and phase separation
• Use of PCM reduces the energy storage volumes compared to SHS mediums.
• SHS can be used for low temperature applications while PCM is recommended for high temperature applications.
• Use of SHS in powder form will be highly advantageous as thermal diffusivity will be high.
• PCM’s having low melting point will have lower life cycle as the number of charging and discharging cycles will be high.
• In indirect solar cooker, TES is efficient and safe to use. However, from economic point of view, the use of TES has been found viable for community cooking.
• The composite of SHS and LHS could be a potential area for research in order to improve the performance economically.
• The method of utilization of energy storage mediums also affects their thermal performance.
• Use of HTF can also contribute to the improvements of the performance of solar cooking units, especially with indirect cooking units.
• Various geometrical and design parameters of the cooking vessel and energy storage unit can play a significant role in better output.

## Data Availability

All data underlying the results are available as part of the article and no additional source data are required.
