# Supplementary material for: Analysis of energy storage materials for developments in solar cookers
Source: F1000Res. 2023 Feb 21;11:1292. Originally published 2022 Nov 11. [Version 2] doi: 10.12688/f1000research.126864.2 (PMC10175959; doi:10.12688/f1000research.126864.2)
Supplement: Peer review history

# Peer review: Reviewer response for version 2

**DOI:** 10.5256/f1000research.144549.r164093

**Published:** 2023-05-11

**Authors:** Katlego A. Lentswe

**Affiliations:** 1 Department of Physics and Electronics, Material Science, Innovation and Modelling (MaSIM) Research Focus Area, North-West University, Mmabatho, South Africa

Approved

Is the work clearly and accurately presented and does it cite the current literature?

Partly

If applicable, is the statistical analysis and its interpretation appropriate?

Not applicable

Are all the source data underlying the results available to ensure full reproducibility?

Yes

Is the study design appropriate and is the work technically sound?

Yes

Are the conclusions drawn adequately supported by the results?

Yes

Are sufficient details of methods and analysis provided to allow replication by others?

Yes

Reviewer Expertise:

Solar cooker with thermal energy storage systems

I confirm that I have read this submission and believe that I have an appropriate level of expertise to confirm that it is of an acceptable scientific standard.

# Peer review: Reviewer response for version 2

**DOI:** 10.5256/f1000research.144549.r164094

**Published:** 2023-05-04

**Authors:** Brian Norton

**Affiliations:** 1 International Energy Research Centre, Tyndall National Institute, University College Cork, Cork, Ireland

The authors' revision of the paper successfully address the comments of reviewers.

Is the work clearly and accurately presented and does it cite the current literature?

Partly

If applicable, is the statistical analysis and its interpretation appropriate?

Not applicable

Are all the source data underlying the results available to ensure full reproducibility?

No source data required

Is the study design appropriate and is the work technically sound?

Yes

Are the conclusions drawn adequately supported by the results?

Partly

Are sufficient details of methods and analysis provided to allow replication by others?

Yes

Reviewer Expertise:

NA

I confirm that I have read this submission and believe that I have an appropriate level of expertise to confirm that it is of an acceptable scientific standard.

# Peer review: Reviewer response for version 1

**DOI:** 10.5256/f1000research.139317.r155692

**Published:** 2022-12-07

**Authors:** Katlego A. Lentswe

**Affiliations:** 1 Department of Physics and Electronics, Material Science, Innovation and Modelling (MaSIM) Research Focus Area, North-West University, Mmabatho, South Africa

1. The author could rephrase the topic from “Analysis of energy storage materials for developments in solar cookers” to “Analysis of energy storage materials for solar cookers”
2. For Figure 1 I suggest that the author use a different figure similar to that used in “Latent thermal energy storage technologies and applications: A review” by Jouhara et al.
3. On the abstract the following sentence “Primarily, oils and pebbles are most commonly used as sensible heat storage (SHS) while organic phase change materials (PCMs) are used as latent heat thermal energy storage materials (LHTES).”  Can be modified I suggest the author must also include water because it has been utilized extensively over the years.
4. “Solar thermal energy systems have higher efficiencies, but they lack in the aspect of energy backup.” I suggest the author must use “storage” than backup.
5. On the introduction section the author couldn’t properly link the sentences and paragraphs it was very confusing and not easy to follow.
6. I think the method section was not supposed to be included because this is a review paper, rather a heading that will indicate or show the classification section.
7. This sentence does not sense “TES can be classified under SHS and LHS mediums.” Rather say SHS and LHS mediums are classified under TES.
8. I suggest a major revision because the format of the manuscript is disorganized.

Is the work clearly and accurately presented and does it cite the current literature?

Partly

If applicable, is the statistical analysis and its interpretation appropriate?

Not applicable

Are all the source data underlying the results available to ensure full reproducibility?

Yes

Is the study design appropriate and is the work technically sound?

Yes

Are the conclusions drawn adequately supported by the results?

Yes

Are sufficient details of methods and analysis provided to allow replication by others?

Yes

Reviewer Expertise:

Solar cooker with thermal energy storage systems

I confirm that I have read this submission and believe that I have an appropriate level of expertise to confirm that it is of an acceptable scientific standard, however I have significant reservations, as outlined above.

# Response

**Published:** 2023-02-16

**Authors:** Rahul Khatri

**Affiliations:** Manipal University Jaipur, India

**S. No 1**

**Comment**

 The author could rephrase the topic from “Analysis of energy storage materials for developments in solar cookers” to “Analysis of energy storage materials for solar cookers”

**Response**

 The study was carried out for future developments in solar cookers. However, requested the editor for suggestion.  

**S. No 2**

**Comment**

 For Figure 1, I suggest that the author use a different figure similar to that used in “Latent thermal energy storage technologies and applications: A review” by Jouhara et al.

**Response**

 Figure 1 is cited from the following article,

*“Helm M, Keil C, Hiebler S, et al.: Solar heating and cooling system with absorption chiller and low temperature latent heat storage: Energetic performance and operational experience. Int. J. Refrig. 2009;32:596–606”.* **10.1016/j.ijrefrig.2009.02.010**

**S. No 3**

**Comment**

 On the abstract the following sentence “Primarily, oils and pebbles are most commonly used as sensible heat storage (SHS) while organic phase change materials (PCMs) are used as latent heat thermal energy storage materials (LHTES).”  Can be modified I suggest the author must also include water because it has been utilized extensively over the years.

**Response**

 Modified as suggested.

**S. No 4**

**Comment**

 Solar thermal energy systems have higher efficiencies, but they lack in the aspect of energy backup.” I suggest the author must use “storage” than backup.

**Response**

 The backup word is replaced with storage.

**S. No 5**

**Comment**

 On the introduction section the author couldn’t properly link the sentences and paragraphs it was very confusing and not easy to follow.

**Response**

 The introduction section is modified as per the suggestion

**S. No 6**

**Comment**

 I think the method section was not supposed to be included because this is a review paper, rather a heading that will indicate or show the classification section.

**Response**

 “Methods” heading is replaced by “Organization of the study”

**S. No 7**

**Comment**

 This sentence does not sense “TES can be classified under SHS and LHS mediums.” Rather say SHS and LHS mediums are classified under TES.

**Response**

 Modified as suggested.

# Peer review: Reviewer response for version 1

**DOI:** 10.5256/f1000research.139317.r155694

**Published:** 2022-11-15

**Authors:** Brian Norton

**Affiliations:** 1 International Energy Research Centre, Tyndall National Institute, University College Cork, Cork, Ireland

This paper provides a review of the use of sensible phase change materials for energy storage in solar cookers. Energy storage is required in solar cookers if their use is to be extended to applications where cooking is either in the evening or at night. Solar cookers used at such times are unlikely to be located outdoors so fluids for the transfer of heat from the outdoor to the indoor environment are also summarized. 

 The paper could provide a more comprehensive and complete review of the literature on this topic. In particular on (i) the solar collectors or inverted absorber mirrors used to transfer heat indoors and (ii) the charging/discharging rates of the various heat storage materials when combined with a range of heat transfer enhancements. The conclusions presented need to be linked to specific previous work. Some conclusions seem odd; for example, is it universally true that PCMs with lower melting points have shorter lives as they cycle more often?

 Some conclusions are lacunae, for example "use of PCM reduces storage volumes compared with SHS medium; true but only if the application requires a large amount of heat stored at the phase transition temperature; for heat stored over wide temperature range the difference in volume is less.

 The work should be contextualized more realistically, for example, in bulk PCMs the phase transition takes place over a range of temperatures as unmelted PCM material convects within that already melted. The PCM line in Figure 1 thus does not apply to the bulk materials discussed in this paper.

Is the work clearly and accurately presented and does it cite the current literature?

Partly

If applicable, is the statistical analysis and its interpretation appropriate?

Not applicable

Are all the source data underlying the results available to ensure full reproducibility?

No source data required

Is the study design appropriate and is the work technically sound?

Yes

Are the conclusions drawn adequately supported by the results?

Partly

Are sufficient details of methods and analysis provided to allow replication by others?

Yes

Reviewer Expertise:

Solar energy applications.

I confirm that I have read this submission and believe that I have an appropriate level of expertise to confirm that it is of an acceptable scientific standard, however I have significant reservations, as outlined above.

# Response

**Published:** 2023-02-16

**Authors:** Rahul Khatri

**Affiliations:** Manipal University Jaipur, India

**S.No. **1

**Comment**

 The paper could provide a more comprehensive and complete review of the literature on this topic. In particular on (i) the solar collectors or inverted absorber mirrors used to transfer heat indoors and (ii) the charging/discharging rates of the various heat storage materials when combined with a range of heat transfer enhancements. The conclusions presented need to be linked to specific previous work. Some conclusions seem odd; for example, is it universally true that PCMs with lower melting points have shorter lives as they cycle more often?

**Response**

 The section named “Modified Solar Collectors” is included as per the suggestion.

 The Conclusion is modified/removed as suggested.

**S.No. 2**

**Comment**

 Some conclusions are lacunae, for example "use of PCM reduces storage volumes compared with SHS medium; true but only if the application requires a large amount of heat stored at the phase transition temperature; for heat stored over wide temperature range the difference in volume is less.

**Response**

 The conclusion is modified/removed as suggested.

**S.No. 3**

 The work should be contextualized more realistically, for example, in bulk PCMs the phase transition takes place over a range of temperatures as unmelted PCM material convects within that already melted. The PCM line in Figure 1 thus does not apply to the bulk materials discussed in this paper.

**Response**

 Figure 1 is used to compare LHS and SHS over a wide range of temperatures. Specific melting temperature is considered for PCM in this figure.
